# The Hippo-Yes Association Protein Pathway in Liver Cancer

**DOI:** 10.1155/2013/187070

**Published:** 2013-08-06

**Authors:** Lu Jie, Wang Fan, Dai Weiqi, Zhou Yingqun, Xu Ling, Shen Miao, Cheng Ping, Guo Chuanyong

**Affiliations:** Department of Gastroenterology, Shanghai 10th People's Hospital, Tongji University, School of Medicine, No. 301, Yanchang Road, Shanghai 200072, China

## Abstract

Hepatocellular carcinoma (HCC) is one of the most common malignancies worldwide and the third leading cause of cancer mortality. Despite continuing development of new therapies, prognosis for patients with HCC remains extremely poor. In recent years, control of organ size becomes a hot topic in HCC development. The Hippo signaling pathway has been delineated and shown to be critical in controlling organ size in both Drosophila and mammals. The Hippo kinase cascade, a singling pathway that antagonizes the transcriptional coactivator Yes-associated protein (YAP), plays an important role in animal organ size control by regulating cell proliferation and apoptosis rates. During HCC development, this pathway is likely inactivated in tumor initiated cells that escape suppressive constrain exerted by the surrounding normal tissue, thus allowing clonal expansion and tumor development. We have reviewed evolutionary changes in YAP as well as other components of the Hippo pathway and described the relationships between YAP genes and HCC. We also discuss regulation of transcription factors that are up- and downstream of YAP in liver cancer development.

## 1. Introduction

Human hepatocellular carcinoma (HCC) is one of the most common cancers, with nearly 600,000 deaths each year worldwide. In addition, its incidence increases every year. HCC usually develops in patients with chronic inflammatory liver disease such as viral infection and/or exposure to chemical carcinogens. Surgical reaction and liver transplantation are currently the best curative options to treat HCC. However, recurrence or metastasis is quite common in patients who have had a resection [1].

Hepatocarcinogenesis is a complex process associated with accumulation of genetic and epigenetic changes that occur during initiation, promotion, and progression of the disease. The role of hepatitis B virus (HBV) infection in causing HCC is well established. The risk of developing HCC was 200 times higher among employees who had chronic HBV as compared to employees without chronic HBV. Hepatitis B virus X protein (HBx) plays critical roles in the development of HCC. Zhang et al. found that the expression of YAP was dramatically elevated in clinical HCC samples, HBV infected hepatic cell line, and liver cancer tissues of HBx transgenic mice. Overexpression of HBx resulted in the upregulation of YAP, while HBx-RNA interference reduced YAP expression. YAP short interfering RNA was able to remarkably block the HBx-enhanced growth of hepatoma cells *in vivo* and *in vitro*. Hepatitis C virus (HCV) infection is also associated with the development of HCC. As well as with HBV, the majority of HCV patients with HCC have associated cirrhosis. Cirrhosis from any cause is a risk factor for the development of HCC [2]. 

The growing incidence of HCC has generated intense research interest to understand molecular and cellular mechanisms of the disease with the hope of developing innovative therapeutic strategies [3]. Multiple signal pathways take part in the process of HCC development. To explore molecular and cellular mechanism of HCC and find an effective treatment is an emergency task to retrieve patients suffering from HCC.

 Recently, many researchers have revealed the function of Hippo pathway involved in organize size regulation, cell proliferation, cell death, and tumor development [4, 5]. However, the role of YAP in HCC, which is downstream effector of the Hippo pathway, remains to be studied. In this review, we provide a historical perspective of the Hippo pathway and discuss the regulation of YAP upstream and downstream factors in liver cancer.

## 2. Drosophila and Human Hippo Signaling Pathway

 In 1995, the first Hippo pathway component—Wts—was discovered using genetic mosaic screens in Drosophila [6], and then the Hippo pathway was recognized as a kinase cascade that regulates transcription coactivator Yorkie (Yki). Loss of Hippo signaling in Drosophila leads to tissue overgrowth due to increased cell proliferation and decreased cell death [7]. Yes-associated protein (YAP) and transcriptional co-activator with PDZ-binding motif (TAZ, also called WWTR1) were identified as Yki homologs in mammals [8]. These Yki homologs are phosphorylated and inhibited by the Hippo pathway through cytoplasmic retention [9]. 

The Hippo pathway can also be activated by cell stress and induce apoptosis [10, 11]. The mammalian Hippo pathway includes STE20 family protein (MST) kinases (MST1 and MST2) and large tumor suppressor (LATS) kinases (LATS1 and LATS2) [12]. When the pathway is activated, MST kinases phosphorylate LATS kinases, which phosphorylate the transcriptional co-activators YAP and/or TAZ [13]. A series of recent studies have demonstrated that MST1, MST2, Sav1 (also known as WW45), and YAP genes are important for growth control and tumorigenesis in the liver [14]. 

**Figure 1:**
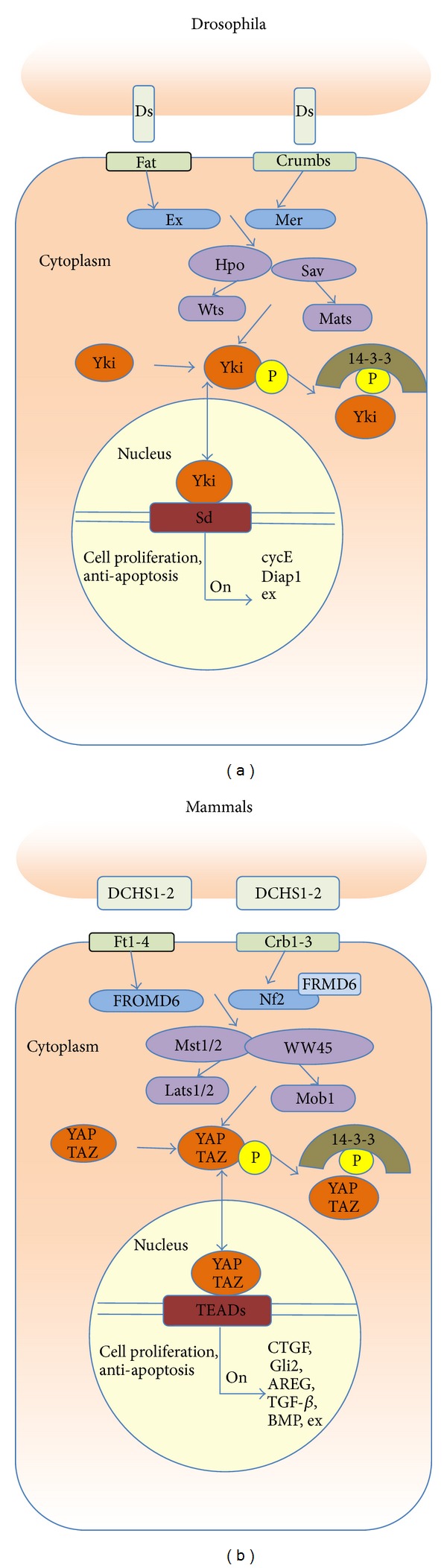
The Hippo pathway in Drosophila (a) and mammals (b). In the Drosophila, the upstream regulation factors exist in both surface membrane, such as Ds, Fat, and Crumbs, and submembrane proteins, such as Ex and Mer. The main effectors of Hippo pathway upstream are Hpo, Sav, Wts, and Mats, and the downstream effectors are Yki and Sd. In the mammals, the homologies of Drosophila in surface membrane protein are DCHS1-2, Ft1-4, and Crb1-3. The submembrane regulators are Nf2 and FROMD6. The Hippo pathway core machinery consists of Mst1/2, WW45, Lats1/2, and Mob1 and downstream effectors are YAP, TAZ, and TEADs. When surface membrane proteins are activated, they will activate subsequence effectors, which will then recruit Hippo core effectors to form complex, such as FRMD6-Nf2 and YAP-TAZ (Yki in Drosophila). Activated Hippo kinase complex will phosphorylate and then be inactivated through interaction with 14-3-3 proteins. Nonphosphorylated YAP/TAZ transfer into the nucleus, interact with TEADs (Sd in Drosophila), and then drive target gene expression to promote cell proliferation and suppress apoptosis.

The Hippo pathway downstream effector is the transcriptional co-activator Yki in Drosophila and YAP/TAZ in mammals, respectively [15]. As shown in Figure 1, each of these genes has an ortholog in the Drosophila Hippo tumor suppressor pathway, which forms a gene network that monitors cell-cell contact and cell polarity, and thereby restricts organ overgrowth [16]. In Drosophila, the Hippo pathway components include Wts, Salvador (Sav), Hippo, and Mats. Together, they form the core Drosophila Hippo pathway in which Hippo kinase, in association with the adaptor protein Sav, phosphorylates and activates Wts kinase, which is associated with an activating subunit Mats (Figure 1) [17]. Yki was identified as a Wts-interacting protein [18].

Some components of the Hippo pathway are highly conserved in mammals such as MST1/2 (the Hippo homolog), WW45 (the Sav homolog; also called Sav), Lats1/2 (the Wts homolog), Mps one binder (Mob1) (the Mats homolog), YAP and its paralog TAZ (both are Yki homologs) [19], and Mer (the Mer homolog; also called Neurofibromatosis factor 2 (NF2)), while others are in a lesser degree such as FRMD6 (the Ex homolog) and FAT4 (the FAT homolog). Deregulation of the Hippo pathway in the mammals often results in tumorigenesis [20]. More strikingly, human YAP, Lats1, MST2, and Mob1 can functionally rescue the corresponding Drosophila mutants *in vivo*, suggesting functional conservation of these proteins across species [21]. 

Until recently, the Hippo pathway was viewed as a straightforward phosphorylation cascade. The upstream kinase Hippo is considered as a member of the Ste20-like family of serine/threonine kinases and likely activated through autophosphorylation or an unknown kinase [1, 22]. Active Hippo can then phosphorylate and activate NDR serine/threonine kinase Wts [8], which in turn phosphorylates transcription co-activator Yki [23, 24]. Phosphorylation inactivates Yki by causing its retention in the cytoplasm by 14-3-3 proteins and thereby preventing its entry into the nuclei and association with its transcription factor partners, such as Scalloped (TEAD in mammals). Yki inactivation results in silencing of its target genes, including progrowth and anti-apoptotic factors such as cyclin E, Drosophila inhibitor of apoptosis-1 (DIAP1), and microRNA bantam [25]. Thus, the Hippo pathway restricts tissue size by antagonizing Yki function [26]. Lats1/2 is phosphorylated by MST1/2 on its activation loop and hydrophobic motif, and it is also possible with auto-phosphorylation involved [27]. Furthermore, YAP/TAZ regulate SMAD2/3 nuclear localization in response to cell density [28, 29].

## 3. YAP and Regulation of Adaptive Liver Enlargement

On a screen for copy-number changes in mouse mammary tumors, YAP is the only gene found in a small 350 bp amplicon from a region, that is, syntenic to a much larger locus amplified in human cancers at chromosome 11q22. Overexpression of human YAP induced epithelial-to-mesenchymal transition (EMT) and suppressed apoptosis, growth factor-independent proliferation, and anchorage-independent growth [30]. YAP was also first cloned as a protein bound to nonreceptor tyrosine kinase (c-Yes) [31].

Growth control in the liver has a number of unusual features compared with other organs. Liver is known for its remarkable capacity to regenerate following a two-third partial hepatectomy (PHx) or injury. Normal adult liver is mainly composed of two parenchymal cell types—hepatocytes and the cholangiocytes [32]. During embryogenesis, a common progenitor cell gives rise to both of these cell types. In the adult, liver cells are largely quiescent dividing approximately once per year. Similarly, in response to various forms of liver injury or partial hepatectomy, liver mass is restored through cell division of remaining parenchymal cells [33]. Dedifferentiated adult hepatocytes, rather than multipotent stem cells, are the source for tissue replenishment and cell turnover in the damaged liver [34]. Components of the Hippo pathway such as MST1 and MST2 have been shown to play important roles in regulating/restricting liver progenitor/stem cells. Therefore, it is foreseeable that the Hippo pathway could be one possible mechanism involved in inhibition of stem/progenitor cell proliferation in mature liver [35]. In YAP transgenic mice, liver cells were switched to a proliferative state leading to an abnormal increase (500%) in the liver/body weight ratio and were also resistant to Fas-mediated apoptosis. Moreover, recent studies also demonstrated that knocking out YAP led to hepatocyte and cholangiocyte injury and loss [36]. 

## 4. Studies Supporting a Role for YAP1 in HCC Development

Liver cancer is the fifth most common cancer worldwide and is the third leading cause of cancer deaths [37]. Risk factors in common understanding are repeated onset of chronic liver damage, chronic viral hepatitis, and inflammation leading to and suggesting repeated cycles of cell injury, death, and regeneration as disease predisposition [38]. However, hepatocarcinogenesis is still a long-term, multistep process involving multiple risk factors and different genetic alterations that ultimately lead to malignant transformation of the hepatocytes [39].

Gene expression analyses have been used to identify genes that are commonly deregulated in different tumor types, such as gastric, breast [40], prostate, and lung cancers [41]. Using mouse models of liver cancer initiated from progenitor cells, YAP and BIRC family (cIAP) 1 are identified as candidate oncogenes in recurrent amplification at chromosome 9qA1, which is the syntenic region of human chromosome 11q22 [42]. Both YAP and cIAP1 accelerated tumorigenesis and were required to sustain rapid growth of amplicon-containing tumors [43]. The YAP gene was also reported to be amplified and overexpressed in other human cancers, such as oral squamous cell carcinoma, primary intracranial ependymomas, malignant pleural mesotheliomas, and oral cancer [44–46]. YAP has been implicated as an oncogene and is altered in different kinds of human digestive system cancers (Table 1), especially hepatocellular carcinoma. Zhao et al. evaluated YAP expression in human HCCs by immunohistochemical staining. They found that among the 115 cases of HCC samples examined, 63 samples (54%) showed strong YAP staining, while 95% of normal liver tissue samples showed very weak staining, suggesting a significant difference in YAP protein levels between normal and cancerous tissues. Other researchers examined the expression level in an HCC cohort in China and found that both the YAP protein and mRNA transcription levels were significantly elevated in the majority of HCC tumorous tissue when compared with adjacent nontumor tissue by 62% to 9%, respectively [47–49]. These results suggest that YAP activation plays an important role in human HCC, and an impaired Hippo pathway might be a common mechanism for YAP activation.

YAP is overexpressed in HCC and has been considered as an independent HCC prognostic marker. Moreover, using a conditional expressed YAP transgenic mouse model, it was shown that YAP overexpression leads to HCC development, which also suggests a direct link between dysregulation of the Hippo pathway and liver tumorigenesis [21] (Table 2).

Furthermore, recent work demonstrated a role of MST1/2 kinases as tumor suppressors because combined deficiency of MST1/2 kinases leads to loss of the inactivation of YAP phosphorylation, massive liver overgrowth, and development of HCC [49]. Regarding liver cancer in human patients, the most compelling information related to YAP is that approximately 50% of human HCC show aberrant overexpression and nuclear localization of YAP [51] and a small fraction of which is attributable to YAP gene amplification [50].

Fernado et al. found that activation of YAP1 for 35 days in adult mice resulted in a more than 4-fold increase in liver size [31]. In addition, expressing YAP1 for 4 days allowed hepatocytes to be unresponsive to Fas-mediated apoptosis. More interestingly, the increase in liver mass could be completely reversible because interruption of YAP1 expression for 5 weeks resulted in a normal size of the liver without any gross abnormalities [52]. 

## 5. Upstream Components of the Hippo Pathway Are Involved in HCC via Regulation of YAP Expression

 The components of the Hippo pathway are made up of tumor suppressors and oncogenes [9]. The Hippo core components and upstream regulators such as NF2 and MST are predominantly involved in tumor suppressor function, whereas TAZ, YAP, and TEADs are involved in oncogenic events [6, 53].

 YAP is a pivotal effector of the pathway. As mentioned before, overexpression of YAP causes EMT, growth factor independent growth, and oncogenesis [12, 49, 51]. The biological activity of YAP was suppressed by phosphorylation at several HXRXXS motifs, which renders cytoplasmic retention via interaction with 14-3-3 proteins [51, 54].

 NF2, the human ortholog of Mer, is a known tumor suppressor. NF2 is structurally similar to members of the ERM family of proteins that are thought to link cytoskeletal components with surface proteins of the plasma membrane [55]. Studies suggest that NF2 interacts with YAP to promote phosphorylation of serine and cytoplasmic retention [46]. Moreover, liver-specific deletion of NF2 in mouse leads to both CC and HCC due to aberrant epidermal growth factor receptor rather than deregulation of the Hippo pathway [56]. These results suggest that there may be multiple mechanisms for NF2 to exhibit tumor suppressor function [57]. 

MST1/2 double knockout mice show liver overgrowth and HCC [21, 22, 35]. This is consistent with the phenotype of transgenic YAP overexpressing mice, which results in liver overgrowth and cancer [49]. Interestingly, MST1 and MST2 are cleaved to shorter and active forms, which are absent in 30% of human HCC. In addition, low YAP phosphorylation was also observed in these HCCs [58]. Upregulation of nuclear YAP and its known target genes, such as connective tissue growth factor CTGF and survivin was observed in oval cells [59, 60]. Mark et al. demonstrated that E-catenin suppressed tumor development in murine epidermis and regulated YAP activity. Therefore, liver cells can adjust their rates of proliferation accordingly to ensure normal tissue homeostasis and protect against tumor development [61, 62].

## 6. Candidate YAP Target Genes and the Mechanism of HCC Development

YAP has been reported to bind and regulate various human transcriptional regulators including p73 and p53-binding protein-2 (p53BP2) [63]. Moreover, some functions of YAP appear different from those of Yki although they are homologs [64]. Therefore, precise biological function and physiological regulation of YAP and/or Yki require further investigation [65].

YAP-TAZ-TEAD complex is downstream effector of the Hippo pathway and the transcriptional coactivator of Yki in the fly [66]. The complex is able to coactivate Runx2-dependent gene transcription and repress PPAR*γ*-dependent gene transcription, which promote mesenchymal stem cells (MSCs) to differentiate between osteoblasts and suppress differentiation of MSCs into adipocytes [67]. The complex has also been documented in regulation of nuclear shuttling of Smads to regulate TGF*β* signaling [68]. Among the four TEADs proteins, TEAD1 and TEAD4 are most often associated with proliferation and cancer development [69]. In addition, TAZ has been demonstrated to interact with the P/LPXY motif at the C terminus of Glis3 to regulate Glis3-mediated gene transcription [70]. Glis3 is a member of the Glis subfamily of Kruppel-like zinc finger transcription factors. This protein functions as both a repressor, and an activator of transcription and is especially in the development of pancreatic beta cells, liver, and kidney. Single nucleotide polymorphisms in Glis3 have been associated with an increased risk of type1 and type2 diabetes, while overexpression of Glis3 is associated with several types of human cancers [71].

## 7. Conclusions

 A better understanding of the mechanisms involved in liver cell proliferation may represent an important approach to develop therapeutic strategy for HCC. The Hippo pathway is emerging as one of the key signaling pathways regulating cell proliferation and apoptosis associated with normal development, stem cell self-renewal, and differentiation [11]. The Hippo pathway is also activated in a cell density-dependent manner and by stress signals such as oxidative stress and irradiation [6]. Although molecular aspects of the Hippo pathway and YAP/TAZ-TEAD effector complex are clearly established, details of the upstream regulators of the Hippo pathway and how they regulate the Hippo core components during development and tissue homeostasis remain elusive. Activation of the mammalian Hippo pathway results in several molecular events; however, phosphorylation and subsequent retention of YAP and TAZ in the cytoplasm is a major consequence [70].

Better definition of HCC molecular pathogenesis could have significant impact on the development of new treatment strategies [1]. The Hippo kinase cascade has been shown to have clearly pathogenic implications in hepatocarcinogenesis; therefore, its regulators might represent novel targets for molecular intervention [68]. Moreover, the Hippo signaling is also important in HCC development in nongenetically manipulated animals, which further support the notion that pathways governing tissue overgrowth and size should be explored as potential therapeutic targets for human HCC.

## 8. Summary

In this review, we provide a historical perspective of the Hippo pathway and discuss the regulation of YAP upstream and downstream factors in liver cancer. This review provides a new notion for Hippo pathway in HCC development and explores a potential therapeutic target for HCC patients.

## Figures and Tables

**Table 1:** Summary of YAP activation in different human digestive system cancers.

Tissue type	Tissue subtype	Negative (%)	Positive (%)
Liver	Normal liver	91	9
		95	5
	HCC	38	62
		46	54

Esophagus	Normal esophagus	61	39
	Adenocarcinoma	54	46
	Metastatic disease	62	38

Colon	Normal colon	67	33
	Neoplastic colon	14	86

Stomach	Normal stomach	86	14
	Gastric adenocarcinoma	70	30
	Gastric metastatic disease	65	35

**Table 2:** Description of the different phenotypes resulting from YAP activation in mice.

Mice	Liver defects	Liver tumorigenesis	Reference
Double transgenic LAP1/tTA-YAP S127A	Hepatocyte proliferation and increased liver size after activation of an inducible YAP transgene (reversible effect)		[50]

Double transgenic ApoE/rtTA-YAP	Hepatocytes are resistant to Fas-mediated apoptosis	Lethal HCCs	[48]

Albumin-Cre YAP c/c	Increased liver size Defects in hepatocyte survival and bile duct development severely impaired		[22]
